# Local agro-industrial by-products offer feasible options to supplement breeding rams in mixed farming systems of Ethiopia

**DOI:** 10.1016/j.vas.2024.100413

**Published:** 2024-11-27

**Authors:** Tesfa Getachew, Bimrew Asmare, Ashraf Alkhtib, Wamatu Jane

**Affiliations:** a Debre Birhan Agricultural Research Center, Debre Birhan, P. O. Box 112, Ethiopia; b Department of Animal Sciences, Bahir Dar University, Bahir Dar, P.O. Box 79, Ethiopia; c School of Animal, Rural and Environmental Sciences, Nottingham Trent University, Nottingham NG25 0QF, UK; d International Center for Agricultural Research in the Dry Areas (ICARDA), P.O. Box 5689, Addis Ababa, Ethiopia

**Keywords:** Flushing, Rams, Agro-industrial by-products, Semen quality, Reproductive characteristics

## Abstract

This study assessed the supplemental effect of flushing Menz breeding rams with local agro-industrial by-products on their reproductive performance and semen quality. In a completely randomized design, rams (*n=* 49) with an initial weight of 25.69+2.8 kg and average age of 18.79±3.9 months were allocated to seven treatments with seven replicates. Supplementation consisted of varying combinations of metabolizable energy (7.02, 8.14, 9.91 MJ ME/kg DM) and crude protein (CP) (8.17 and 12.5 %) levels above the basal diet (pasture grazing). The experimental treatments were control, T0 (8 h of free grazing in natural pasture and fallow lands providing 4.7 MJ/Kg metabolizable energy and 6.65 CP) supplemented with T1 (500 g oats grain), T2 (500 g maize bran), T3 (500 g wheat bran), T4 (300 g oat grain +150 g broken lentil grain), T5 (300 g maize bran + 150 g broken lentil grain), T6 (400 g wheat bran). Supplementation significantly (*P* < 0.05) improve body condition score, scrotal circumference, semen ejaculation volume, spermatozoa concentration, and predictive potential of rams to serve ewes. Notably, all variables within the supplemented groups showed no significant differences (*P* > 0.05), except for scrotal circumference, where T1 and T3 showed a greater increase (*P* < 0.05) compared to other treatments. High and positive correlations (*r* = 0.89) between scrotal circumference and volume of semen were observed. Semen volume and concentration correlated positively (*r* = 0.7 and *r* = 0.9 respectively) with the rams’ potential to serve ewes. It is concluded that the reproductive activity of Menz rams is influenced by their nutritional state. Agro-industrial by-products can supply adequate protein and energy levels to enhance reproductive potential of rams. The studied agro-industrial by-products are relatively inexpensive and easily accessible compared to commercial concentrates. Hence, based on the cost-effectiveness of supplementary feed used; it is recommended that T3 and T6 wheat bran-based supplementation strategies be employed to enhance the reproductive performance, semen quality, and the potential of Menz rams to serve ewes. Further research is needed to evaluate how different seasons affect the levels of protein and energy supplementation required for ram flushing.

## 1 Introduction

The Community-Based Sheep Breeding Program (CBBP) for the Menz sheep was initiated in 2009 to improve sheep productivity through breed selection and to improve the income of smallholder farmers through the selection of genetically superior breeding stock (Gizaw, 2011; Mikena et al., 2012). To accelerate CBBP for Menz sheep, artificial insemination (AI) technique was recently implemented by Debre Birhan Agricultural Research Center in Amhara region, Ethiopia. Artificial insemination is one of the tools in animal breeding programs to improve genetically and economically important traits in animals (Morrell, 2011, Tsuma et al., 2015). For breeding rams, the standard age is 1.5 years or older to achieve a normal ejaculatory volume of 0.8 to 1.2 ml, with an average of 1 ml (Ahmed et al., 2017). Semen volume (ml) and motility (scale 1–5) of Menz breeding rams at 6, 9 and 12 months of age have been reported to be 0.3 & 1.06, 0.47 & 1.62, and 0.58 & 2.65 respectively (Rege et al., 2000). This indicates that the semen volume and other related semen quality parameters of the Menz breed need improvement. Under nutrition, whether due to single or multiple nutrients or energy, as well as nutrient imbalances, results in reduced hormone production and poor semen quality (Brown, 1994). However, very few studies have been carried out on the effect of nutrition on reproductive characteristics, including semen quality and quantity, particularly under tropical conditions.

Poor nutrition results in low reproductive performance of sheep (Abebe et al., 2020). Crope residues and post-harvest stubble grazing contribute up to 100 % of total dry matter intake of sheep especially in the dry season when natural pasture is poor in terms in productivity and quality (Alkhtib et al., 2017). Crop residues are high in fibre and low in digestibility and nitrogen (Devendra & Thomas, 2002). Ethiopian sheep which feed predominantly on crop residues and poor natural pasture do not obtain maintenance requirements of protein and energy (Preston, 1995). Energy and protein supplementation were found to improve ruminant utilization of poor-quality roughages (Lenné et al., 2003). Energy and protein supplementation above maintenance level was reported to improve reproductive performance of sheep (Kearl, 1982). Yet, this practice is still not common among Ethiopian farmers. Some approaches would be to supplement animal diets with the missing nutrients or energy, removing the imbalances, or both. In developing countries, this would be of particularly importance (Martin et al., 2010). Such improvements would help avoid classic cases of diet-related caused, but often reversible, subfertility or infertility. Flushing is a temporary but purposeful increase in nutritional levels during the breeding season of livestock. Flushing should be done 4–6 weeks before mating and during mating (Allaoui et al., 2018). Optimal dietary supplementation above farmers’ traditional practices that maximize the reproductive performance of rams should be determined. However, to date, the influence of flushing on the reproductive performance of rams has not been well documented in the Central Highlands of Ethiopia.

There is an increasing trend towards the use of conventional agro-industrial by-products as energy and protein supplements. The use/adoption of agro-industrial byproducts is crucial for the intensification of livestock feeding systems in developing countries. Typical agro-industrial by-products in the central highlands of Ethiopia include maize bran, wheat bran, lentil screenings and oat grains. Ethiopia produced 10,022,000 t of maize grains in 2020 (CSA, 2021). Maize grains are considered the most important staple food for Ethiopians in terms of calorie intake (Berhane et al., 2011). Wet milling of maize grain produces large quantities of a fibrous fraction called maize bran, which could be estimated at 1,500,000 tons annually (Singh et al., 2013). Maize bran was used as an energy supplement in high-roughage diets to lambs and steers (Oliveros et al., 1989). Maize bran was introduced to small ruminant diets in a level up to 25 % (Katongole et al., 2009). Goats fed a diet of Rhodes grass and *Gliricidia sepium* hay had better performance and nutrient digestibility when supplemented with maize bran (Ondiek et al., 1999). The annual production of wheat bran was 750,809 tone DM per year (Feyisa et al., 2024). Lentil grains are a very important part of the daily meal of Ethiopians (Erksine et al., 2009). Lentil grain production in Ethiopia was 113,018 t in 2020 (CSA, 2020). Lentil screenings are a by-product of cleaning lentil seeds, and are a mixture of lentils, chaff, dust, weed seeds, and cereal grains. Lentil grain screening produces a considerable amount of residue, including grain, straw, and foreign matter (weed grains, and dust) (Stanford et al., 1999). Lentil screening is relatively inexpensive and can be a useful source of protein and energy supply for ruminants. Lentil screenings have high levels of crude protein (22 %−25.9 %), low level of neutral detergent fiber (20.9 %), and a high level of metabolizable energy (12.8 MJ/kg) (Joshi et al., 2017). Oats are very important feeds as they provide excellent energy, adequate in fibres which are slowly fermented in the reticulo-rumen of sheep (Sormunen Cristian, 2013). The effect of Menz ram supplementation with agro-industrial by-products and oats grains on reproductive performance is not studied. Thus, the current study aims to determine the effect of flushing rams with agro-industrial by-products and oats grain, with a focus on optimizing protein and energy levels.

## 2 Materials and methods

### 2.1 Description of the study area

The study was conducted on-farm in Menz Mama District. The District covers 67,072 ha of land, lies at an altitude of 1590–3413 m above sea level and receives an average annual rainfall of 896 mm and temperature of 15 to 20 °C, respectively. The area is characterized by a crop-livestock mixed farming system, with sheep being the predominant livestock. The main feed resources available include crop residues, hay collected mainly from natural pastures and fallow lands, grazing of natural pastures, cut and carry of crop weeds, improved forages (oats, vetch, and tree lucerne) and agro-industrial by-products such as, lentil screenings, wheat bran, maize bran, noug seedcake and local beverage residues and oat grain. The current experiment was conducted at the end of main rainy season October and November for a period of five weeks including one week of feed adaptation.

### 2.2 Experimental animals and their management

Menz breeding rams (*n* *=* 49), with an average age of 18.79±3.9 (mean + SD) months, and bodyweight range of 25.69±2.8 (mean + SD) kg, were used in a completely randomized design with seven treatments, each with seven replicates. The rams were treated against internal and external parasites using anti-helminths, Albendazole (7.5 mg/kg weight ingested through the mouth) and Ivermectin (0.2 mg/kg weight, administered through subcutaneous injection), respectively as per recommendations of the Ministry of Livestock and Fisheries, Ethiopia. All rams were adapted to the experimental natural pasture and individual cages for 15 days. On day 16 to the end of the trial, the experimental rams were randomly allocated into 7 treatments, control, T0 (8 h of free grazing in natural pasture and fallow lands providing 4.7 MJ/Kg metabolizable energy and 6.65 CP), T1 (control + 500 g oats grain), T2 (control + 500 g maize bran). T3 (control + 500 g wheat bran), T4 (control + 300 g oat grain +150 g broken lentil grain), T5 (control + 300 g maize bran + 150 g broken lentil grain), T6 (control + 400 g wheat bran) as shown in Table 1. All sheep were offered free grazing for 8 h (9:00–12:00 h and 14:00–17:00 h) and *ad libitum* access to water. The experiment was approved by the Animal Care (No. IACUC-RC2015–07) of the International Livestock Research Institute and the animals were under constant observation by a veterinarian.

**Table 1:** Ingredients and chemical composition of experimental diets.

Treatments	Oat grain (g)	Wheat bran (g)	Maize bran (g)	Broken lentil grain (g)	Natural pasture	Level of energy (MJ ME/kg DM)	CP (% DM)
T0/FP/C					grazing 8h	4.7	6.65
T1	500	0	0	0	grazing 8h	9.93 (50%)	8.4 (H-ME, L-CP)
T2	0	0	500	0	grazing 8h	7.01 (30%)	8.01 (L-ME, L-CP)
T3	0	500	0	0	grazing 8h	9.89 (50%)	12.77 (H-ME, H-CP)
T4	300	0	0	150	grazing 8h	7.03 (30%)	12.1 (L-ME, H-CP)
T5	0	0	300	150	grazing 8h	8.08 (40%)	8.1 (M-ME, L-CP)
T6	0	400	0	0	grazing 8h	8.20 (40%)	12.2 (M-ME, H-CP)

FP/C, farmer practice/ control; T, treatment; H, high; L, low; M, medium; ME, metabolizable energy; CP, crude protein, h=hour.

The DM, CP, ash, NDF, ADF, and ADL contents of the experimental ingredients and natural pasture hay are given in Table 2. Natural pasture hay had lower CP and higher NDF and ADF content than the supplemented feeds.

**Table 2:** Chemical composition of the experimental feeds on DM basis.

Ingredients	DM (%)	Ash (%)	CP (%)	NDF (%)	ADF (%)	ADL (%)	ME (MJ/kg)	IVOMD (%)
Wheat bran	92.18	5.51	15.83	44.30	13.59	3.60	10.7	69.22
Broken lentil screenings	92.53	12.17	23.85	34.42	27.96	8.95	10.4	62.80
Maize bran	91.71	1.48	11.9	44.88	14.23	4.21	13.7	61.65
Oats grain	92.65	5.17	10.2	51.10	24.18	4.73	11.7	55.06
Natural pasture	93.84	10.52	6.87	66.99	43.88	7.52	6.8	49.76

ADF=Acid detergent fiber; ADL=Acid detergent lignin; CP=Crude protein; DM=Dry matter; NDF=Neutral detergent fiber; OM= Organic matter; IVOMD = *Invitro* Organic matter digestibility.

### 2.3 Data collection and analysis

#### 2.3.1 Body weight and body condition score

Live weight was measured gravimetrically using a portable spring balance (Camry, NTB, Camty company), with a capacity of 100 kg and an accuracy of 50 g. The scale was calibrated with standard weights. Ten sheep were then weighed in three replicates to confirm the reliability of the liveweight measurements. Body condition score (BCS) and live weight were recorded at the start of the trial and on a weekly basis thereafter. Body Condition Score of rams was measured by physical examination of fat cover in the backbone and scored on a scale, typically from 1 to 5, with 1 being extremely thin and 5 being excessively fat. Live weight was taken before morning feeding after overnight fasting. This was done by taking the average weight of two consecutive days of overnight fasting. Average daily weight gain (ADG) was calculated as the difference between final and initial body weight divided by the number of days. The body condition score of the rams was assessed on a scale of 1 to 5 (ESGPIP, 2008).

#### 2.3.2 Reproductive performance

Scrotal circumference (SC) and libido character were measured. The SC was measured by firmly pulling the testicles down into the lower part of the scrotum and placing a measuring tape around the widest point. The SC was measured at the beginning and end of the experiment. Libido was assessed according to the protocol as described by Evans and Maxwell (1987) at semen collection and scored on a scale of 1 to 5 (1= very poor and 5=excellent).

#### 2.3.3 Semen quality

Semen quality such as ejaculation volume, the color of semen, sperm mass motility, the concentration of spermatozoa, and morphology of spermatozoa were evaluated. After training the rams for artificial vagina (AV), semen was collected weekly from every ram, early in the morning (7:30 – 8:30am) by using AV (water, 40 °C) and we placed it in a neck clamp. Semen volume was measured using a graduate tube and recorded for every ejaculation. Sperm mass motility was measured using a phase-contrast microscope and scored from zero to five grade scale based on the intensity of wave motion as described by Evans and Maxwell (1987) (Zero= all spermatozoa are immotile (motionless), 1=Very poor; very few spermatozoa are active about 10 % (weak movement around), 2=Poor (some movement of semen was visible about 20–40 % of spermatozoa were live but poor motility),3=Fair (small, slow moving wave 40–70 % of sperm cells were active, 4= Good (dense, vigorous wave movement 75–90 % of sperm cells were active, 5= Very good (cloudy, dense and rapidly moving waves >90 % of spermatozoa were active.

The proportion of morphologically normal and abnormal spermatozoa was also determined by A drop of Eosin, four drops of nigrosine and a drop of semen were placed on a clean, grease free slide then mix the semen first with eosin and then immediately with nigrosine stain. Again, the mixture was taken on the edge of a slide and pulled across the top of another slide leaving a smear, thereafter it was air dried. 200 numbers of spermatozoa were counted under oil immersion at a magnification of 100X in different areas of smear and classify them as normal, head abnormal, mid piece abnormal and tail abnormal sperm (Rege *et al*., 2000).

$$\text{Head}\;\text{abnormal}\;\text{sperm}\;\text{percentage}=\frac{\text{Total}\;\text{head}\;\text{abnormal}\;\text{counted}}{\text{Total}\;\text{numbers}\;\text{counted}\;}\;*100$$

$$\text{Midpiece}\;\text{abnormal}\;\text{sperm}\;\text{percentage}=\frac{\text{Total}\;\text{midpiece}\;\text{counted}}{\text{Total}\;\text{number}\;\text{counted}}*100$$

$$\text{Tail}\;\text{abnormal}\;\text{sperm}\;\text{percentage}=\frac{\text{Total}\;\text{tail}\;\text{abnormal}\;\text{counted}}{\text{Total}\;\text{number}\;\text{counted}}\;*100$$

Viscosity was observed in a laboratory environment by first allowing the semen to liquefy. When the liquefaction was completed, the sample was allowed to drop by the force of gravity through a pipette. The length of the thread was measured as the semen dropped. The normal semen was left with a very small trailing thread and semen with abnormal viscosity left a thread >2 cm long. Viscosity was measured using 4 grades (4= very thick, 3=thick, 2=thin, and 1=very thin). The color of semen was measured by physical observation using 5 grades scales (5=thick cream, 4=creamy white, 3=thin creamy,2=milky, and 1=watery).

#### 2.3.4 Potential of rams to serve ewes

The potential of rams to service ewes (PRSE) was measured within a mating cycle. PRSE predictions were calculated by multiplying semen volume, sperm concentration, and sperm motility, and then dividing by the dosage of artificial insemination (Kurnia et al., 2020). The dosage for artificial insemination was administered according to Menchaca et al. (2005). For cervical insemination with fresh semen:

$$PRSE=\frac{Semen\;volume*\;sperm\;concentration*\;sperm\;motility}{AI\;dosage\;(200000000\;spermatozoa\;per\;0.25\;ml\;straw}$$

#### 2.3.5 Chemical analysis of the experimental feeds

After oven-drying at 100 °C for 24 h, samples were ground to pass through a 1-mm sieve mesh. The samples were analyzed using conventional wet chemistry method. Dry matter (DM) and crude protein (CP) were analyzed according to the methodology of (Cunniff, Patricia & Washington, 1997). Dry matter was determined by oven drying at 105 °C overnight (method 934.01). Ash was determined by burning in a muffle furnace at 500 °C overnight (method 942.05). Nitrogen content was determined by Kjeldahl method using Kjeldahl (protein/nitrogen) Model 1026 (Foss Technology Corp.), (method 954.01). A conversion factor of 6.25 was used to convert nitrogen to crude protein. Neutral detergent fiber, acid detergent fiber (ADF) and lignin were determined as described by Van Soest, Peter and Robertson (1985). Neutral detergent fiber (NDF) did not involve use of heat stable amylase and the result was expressed exclusive of residual ash. Acid detergent fiber was expressed without residual ash. Lignin was determined by solubilisation of cellulose with sulphuric acid. The chemical analyses of the feed samples were carried out at the Animal Nutritional Laboratory of the International Livestock Research Institute (ILRI) in Addis Ababa.

*In vitro* organic matter digestibility was measured in rumen microbial inoculum using *in vitro* gas production technique. The buffer solution was prepared according to the method described by Menk et al. (1979). Rumen fluid was collected prior to morning feeding using a vacuum pump from three ruminal cannulated cows fed a total mixed ration of grass hay (790 g/kg), wheat bran (203 g/kg), salt (3.2 g/kg) and a mineral and vitamin mixture (4.6 g/kg) on a DM basis. The rumen fluid from the cows was composited (1:1, v/v), filtered through four layers of cheesecloth, and added to the buffer solution (1:2, v/v), which was maintained in a water bath at 39 °C under continuous flushing with CO2. The buffered rumen fluid (30 ml) was pipetted into 100 ml syringes containing 0.2 g of sample and immediately placed into a water bath at 39 °C. Gas production was recorded after 24 h of incubation and used to calculate invitro organic matter digestibility (IVOMD) equations as follows:

IVOMD (g/kg) = 14.88+0.889GP+0.45CP+0.0651XA

Where GP: 24 h net gas production (ml/200 mg); CP: Crude protein (g/kg DM); XA: Ash content (g/kg DM). ME(MJ/kg)=TDN(%)*0.15104

### 2.4 Data analysis

Analysis of variance was analysing the study data according to the following models:

Model 1: Live weight, body condition score and scrotal circumference:

Y_(i)_ = M + TRT_(i)_ + *E*_(i)_

Model 2: Semen characteristics:

Y_(ijk)_ = M + *R*_(i)_ + REP_(j)_ + TRT_(k)_ + *E*_(ijk)_

Where Y is the response variable, M is the overall mean, R is the effect of ram, REP is the effect of day of semen collection, TRT is the effect of dietary treatment, and E is the residual. Least square means were compared using Fisher's LSD method at *P* = 0.05. All data analyses were done using R (R Core Team, 2017) computer programming.

## 3 Results

### Body weight change and body condition score of rams

The initial body weight (IBW), final body weight (FBW), average daily body weight gain (ADG), and initial body condition score were similar between supplemented and control groups (Table 3). The current result shows that short-term flushing (4 weeks) of Menz rams with different energy and protein level feed had no effect on body weight change compared to the farmer's practice (natural pasture grazing). Supplemental diets improved (*P* < 0.05) the final body condition score (FBCS). FBCS did not differ (*P* > 0.05) across supplemental treatments.

**Table 3:** Body weight change and body condition score of Menz rams supplemented with agro-industrial by-products providing varying energy and protein levels.

Treatments									
Parameters	T1	T2	T3	T4	T5	T6	T0/Control	P-value	SEM
IBW (kg)	26.98	24.62	27.07	25.2	26.22	22.9	26.87	0.25	2.8
FBW (kg)	29.88	27.00	30.4	28.78	29.3	27.24	27.7	0.4	3.44
ADG (g/d)	138.1	107.74	99.21	170.75	74.29	160.95	138.1	0.34	3.2
IBCS	2.81	2.78	2.77	2.84	2.70	2.71	2.71	0.16	0.45
FBCS	3.78^a^	3.57^ab^	3.91^a^	3.78^a^	3.83^a^	3.6^ab^	2.8^b^	0.049	0.12

LS Means with different superscripts in the same row differ significantly differ at *P* < 0.05; ADG=average daily gain; FBW=final body weight; IBW=initial body weight; IBCS= Initial body condition score, FBCS= Final body condition score; SEM= Standard error of mean.

### Reproductive characteristics of Menz rams

Table 4 shows scrotal circumference (SC) and libido of Menz breeding rams supplemented with different energy and protein levels. The result shows that a 50 % energy supplementation above farmers’ practice (T0) with both T1 (8.4 % CP) and T3 (12.77 % CP) resulted in a significantly larger (*P* < 0.05) final scrotal circumference (FSC) of Menz breeding rams compared to the control. Supplementation above farmers’ practice (T0) had no effect on libido characteristics.

**Table 4:** Reproductive characteristics of Menz breeding rams supplemented with agro-industrial by-products providing varying energy and protein levels.

Treatments									
Parameters	T1	T2	T3	T4	T5	T6	Control	p-value	SEM
ISC (cm)	26	25	25.42	25.14	25	24.21	24.57	0.78	1.8
FSC (cm)	28.14^a^	25.71^b^	27.9^a^	26.9^ab^	26.9^ab^	26.4^ab^	25.35^b^	0.02	1.47
Libido (1–5)	4.7	5	5	5	5	5	5	0.11	0.31

LS Means with different superscripts in the same row differ significantly at *P* < 0.05; ISC = Initial scrotal circumference, FSC= Final scrotal circumference, SEM= Standard error of mean.

### Semen quality of Menz breeding rams

Semen quality evaluation parameters such as semen ejaculation volume, mass sperm motility, spermatozoa concentration (1 × 10^9^), color, and consistency (viscosity) are presented in Table 5. Supplementation (T1-T6) with energy and protein significantly increased semen volume (*P* < 0.001) and concentration of spermatozoa (*P* < 0.05). However, semen volume and spermatozoa concentration did not differ among treatments. Supplementation tended to increase (*P* > 0.05) sperm mass motility, color, and viscosity of semen. The effect of season on sperm volume and quality has not been assessed in the current study but the study of (Alkhtib et al., 2017) showed that photoperiod has its own impact on the reproduction capacities of rams.

**Table 5:** Semen quality of Menz breeding rams supplemented with agro-industrial by-products varying in energy and protein levels.

Treatments									
Parameters	T1	T2	T3	T4	T5	T6	Control/ FP	p-value	SEM
Volume (ml)	0.81^a^	0.66^a^	0.73^a^	0.70^a^	0.76^a^	0.74^a^	0.37^b^	0.0003	0.02
Mass motility (1–5)	4.71	4.71	4.00	4.14	4.4	4.5	4.14	0.34	0.71
Concentration (X10^9^)	1.44^a^	1.40^a^	1.38^ab^	1.46^a^	1.38^ab^	1.66^a^	1.11^b^	0.045	0.08
Color	CW	CW	CW	CW	CW	CW	CW	–	–
Viscosity (1–4)	3	2.87	2.66	2.85	3	3	2.57	0.23	0.35

LS Means with different superscripts in the same row differ significantly at *P* < 0.05; SEM= Standard error of Mean. Color (1–5 grades): 1 = watery, 2 = milky, 3 = thin creamy, 4 = creamy white; 5= thick creamy, Mass Motility (1–5 grades): 1= no perceptive motion, 2 = weak motion without forming any waves, 3 = small, slow-moving waves, 4 = vigorous movement with moderately rapid waves and eddies, 5 = dense, very rapidly moving waves. Viscosity (4=very thick, 3=thick, 2= thin, 1= very thin).

### Sperm morphology

The effect of treatments (T1-T6) on sperm morphology of Menz breeding rams is presented in Table 6. The result shows that supplementation had no significant difference (*P* > 0.05) on sperm morphology. However, there was a tendency (*P* > 0.05) towards lesser abnormality with treatments with higher ME (50 %) and lesser protein (8 %) (T1, T5).

**Table 6:** Spermatozoa morphology of Menz breeding rams fed on grazing natural pastures and supplemented with feeds of different energy and protein levels.

Treatments									
Parameters	T1	T2	T3	T4	T5	T6	T0 (control)	P-value	SEM
Morphology									
Normal (%)	94.42	91.71	91.83	90.85	92.8	88.3	91.28	0.83	6.3
Abnormal (%)	5.57	8.28	8.16	9.14	7.2	12.16	8.71	0.55	4.0
Types of abnormality									
Head	0	0	0	0	0	0	0		
Tail	5	7.85	6.5	8.28	6.8	5.16	8.0	0.63	4.00
Mid-piece	0.57	0.0.57	1.66	0.85	0.4	1.2	0.85	0.64	1.12

LS Means with different superscripts in the same row differ significantly at *P* < 0.05; SEM= Standard error of Mean.

### The potential of Menz rams to serve ewes

The result of the potential of Menz rams to serve ewes fed on grazing natural pastures and flushed with different levels of energy and protein feeds is presented in Table 7. The result shows that there is a significant difference (*P* < 0.05) among supplemented (T1-T6) and control group on the potential of Menz rams to serve ewes. However, there was no significant difference (*P* > 0.05) among the supplemented groups. Lower protein supplementation (T1, T2, T5) tended to show (*P* > 0.05) higher potential to serve, indicating a lower level of protein and energy supplementation can be used for flushing Menz rams.

**Table 7:** Predictive potential of Menz rams to serve ewes fed on grazing natural pastures and supplemented with different levels of energy and protein feeds.

Variables	Treatments							P-value	SEM
	T1	T2	T3	T4	T5	T6	T0(FP)		
Ewes served (head)	27.71^a^	29.09^a^	20.8^a^	22.99^a^	25.96^a^	21.39^a^	10.79^b^	0.0019	2.13

LS Means with different superscripts in the same row differ significantly at *P* < 0.05; SEM= Standard error of Mean.

### Correlation between body condition, reproductive performance and semen characteristics of Menz rams

The correlations between body condition, reproductive performance and semen quality of Menz rams are presented in Table 8. The SC is highly and positively correlated to semen ejaculation volume (*r* = 0.89; *P* < 0.001) and moderately correlated to BCS (*r* = 0.57; *P* < 0.01). BCS moderately influenced semen ejaculation volume (*r* = 0.54; *P* < 0.01) and potential to serve ewes (*r* = 0.44; *P* < 0.05). Potential to serve ewes is highly correlated to semen volume (*r* = 0.9; *P* < 0.001) and spermatozoa concentration (*r* = 0.7; *P* < 0.001). Viscosity is highly correlated to the colour of the semen (*r* = 0.8; *r* < 0.05), but moderately correlated to ejaculation volume (*r* = 0.5; *P* < 0.01) and spermatozoa concentration (*r* = 0.6, *P* < 0.001).

**Table 8:** Correlation matrix between semen characteristics of breeding Menz rams fed on grazing natural pastures and supplemented with different levels of energy and protein feeds.

	BCS	BW	SC	LIB	CON	VOL	COL	PPRSE	VIS	MOT	NOR	ABN
BCS	1	0.44*	0.57**	0.01	0.18	0.54**	0.18	0.44*	0.21	0.29*	0.22	−0.22
BW		1	0.3*	−0.08	0.07	0.13	0.16	0.1	0.14	0.02	−0.07	0.07
SC			1	−0.02	0.16	0.89***	0.24	0.4*	0.2*	0.28	0.21	−0.21
LIB				1	−0.04	−0.022	−0.07	−0.02	−0.08	0.01	−0.18	0.18
CON					1	0.49**	0.53**	0.7***	0.6***	0.14	0.15	−0.15
VOL						1	0.45*	0.9***	0.5**	0.2*	0.22	−0.22
COL							1	0.5**	0.8***	0.35*	0.13	−0.13
PRSE								1	0.56**	0.27	0.21	−0.21
VIS									1	0.3*	0.12	−0.12
MOT										1	0.01	−0.01
NOR											1	−0.68**
ABN												1

*=*p* < 0.05, **=*p* < 0.01, and *** =*p* < 0.001 BCS = Body Condition score, BW = Body Weight,SC = Scrotum Circumference, LIB = Libido, CON = Concentration, VOL = Volume, COL = Color, PRSE = potential of rams to serve ewe, VIS = Viscosity, MOT = Motility, NOR = Normal, ABNO = Abnormal.

## 4 Discussion

Feeding sheep poor quality roughages for long time would cause nutrient deficiency. Ethiopian farmers depend mainly on natural pasture and crop residues for sheep nutrition during mating season (Alkhtib et al., 2017). The natural pasture in the current study contains almost 70 g/kg of crude protein, which is the minimum level required to maintain optimum function of the rumen (Van Soest, 1994). Thus, low reproductive performance of sheep is expected under such conditions. Accordingly, introducing the concept of nutritional flushing is required to enhance reproductive performance of sheep (A.H Al-Haboby et al., 1999). The current study evaluated ram flushing using locally available agro-industrial by-products to improve reproductive performance. This study provides evidence those agro-industrial by-products providing protein and energy at varying levels can beneficially modulate reproductive characteristics and improve semen quality of rams.

Supplementation had no effect on the ADG of the experimental rams. This might be due to the experiment duration (short period, 28 days). However, final body condition score (FBC) varied significantly between the treatment and control group. This might be because of the variation in the crude protein and metabolizable enery content of the feed between the supplement feed and the control or farmers practice. The final body condition scores (FBCS) of the supplemented groups in the current study were comparable with the values reported by Goshme et al. (2020) for Menz breeding rams under on-station management supplemented with commercial concentrate. Furthermore, body condition is directly related to reproductive performance of rams (Maquivar et al., 2021). The observed mean FBCS (3.745) in this study for different levels of energy and protein in supplemented groups was greater than the value recommended for breeding rams (3.5; on a scale of 1 to 5; (NADIS, 2020). This suggests that supplementation of grazing rams with protein and energy prior to breeding time enhances BC of rams. Ensuring adequate ram condition and nutrition prior to breeding allow rams to have enough body condition to remain strong and healthy.

Scrotal circumference is an indicator of male fertility and serving capacity. Rams with normal, large scrotal circumference produce more and higher quality semen than rams of the same age and breed with small diameter (Rekik, Mourad, 2016). Daughters from sires with a larger testicle circumference are more fertile than females sired by males with a smaller circumference. The result of the present study shows that a 50 % energy supplementation above farmers ‘practice with both low (8.4 %) and high (12.77 %) CP resulted in a significantly larger (*P* < 0.05) scrotum circumference of Menz breeding rams compared to the control. The results also showed that the above maintenance energy and protein supplemented groups (30 % and 40 % ME) had no significant difference (*P* > 0.05) with 50 % energy supplementation and also with the control/farmers practice on scrotum circumference. Lack of difference among energy and protein supplemented groups might be the requirement for protein and energy might be satisfied at the lower level of supplementation for scrotal growth.

The greater SC for lower-level supplementation might be due to the optimum utilization of dietary protein (Negesse et al., 2001) and the utilization of Adenosine Triphosphate (ATP) to convert excess ammonia to urea. The results of the supplemented groups in the present study were in line with (Rekik, Mourad, 2016) who stated that the average scrotal circumference increases from 25 cm at 1 year of age to nearly 30 cm at 4 years of age in Ethiopian sheep breeds (Horro, Bonga and Menz). For larger breeds like Awassi, the target SC is 36–38 cm. Scrotal circumference was significantly improved (*P* < 0.05) by supplementation in this study (*P* < 0.05). This agrees with (Ghorbankhani et al., 2015) who found that Sanjabi ram lambs fed high-quality feed had better reproductive organs, particularly the testicular circumference, a trait associated with semen quality.

Semen volume is one of the important factors in semen evaluation and reproductive performance in rams (Ax et al., 2000). This study result indicates that there was no significant variation of different supplement groups on semen volume of rams. However, there was significant improvement on semen volume of rams between supplement and control groups. The supplemented group improve semen volume of rams by more than double. This was due to feeding a higher energy diet increases semen production and quality. The additional energy signals to the ram's body to upsurge gonadotropin releasing hormone (GnRH), which begins a cascade of signals that initiate semen production. Therefore, the current result revealed that supplementation of protein and energy above farmers practice improves semen volume.

The semen volume in this study was greater than the value of 0.15–0.65 ml reported for Ethiopia highland sheep rams (Gebreslassie, 2012). However, the semen volume of the supplemental groups in the present study was comparable to Goshme et al. (2020) who reported a mean semen volume of 0.7 ml value for Menz rams supplemented with commercial concentrate, (Gunawan et al., 2020) who reported 0.71 ± 0.15 ml for (Nasrin et al., 2012) who reported 0.7 ml for Garut Ram rams. This is within the physiological range for rams (Azizunnesa et al., 2014). In contrast, the semen volume observed in this study was lower than that reported by (Jiménez-Rabadán et al., 2016) for Bangladeshi rams, which were 3.4 ml and 6.2 ml for the concentrate supplemented and control groups, respectively. The difference may be due to the influence of genetics, environmental factors or feeding practices. Semen volume is also affected by semen collection methods and collection frequency (Khalifa et al., 2013).

The mean sperm cell concentration of supplemented groups (1.450 × 10^6^ sperm/ml) was significantly higher (*P* < 0.05) than the control in this study (1.11 × 10^9^ sperm/ml). Flushing of Menz rams with energy and protein above farmers practice improves the reproductive performance of breeding rams, however, increasing the level of supplementation did not bring any advantage related to sperm concentration. The sperm cell concentration result of the present study was lower than 2000 - 3500 × 10^6^ sperm/ml (Kaya et al., 2002) reported for Bangladesh rams. Differences in sperm cell concentration may be attributed to frequency of ejaculation, and/or breed type (David et al., 2015). Mass sperm motility is a reliable measure of fertility in rams (Ahemen et al., 2011). This study shows that sperm cell mass motility of Rams was not affected by nutritional flushing this means there was no significant different observed between the control and supplemented groups. The mean mass motility (grade 4) observed in this study is comparable to earlier reports by (Nel-Themaat et al., 2006) in West African dwarf rams in Nigeria, (Talbi et al., 2010) in Gulf Coast native rams, (Vishal, 2014) in Boujaad rams and (Cunha et al., 2012) in Nellore Jodephi rams. As observed in the present study, enhanced levels of protein and energy supplementation compared to the control did not affect semen mass motility. In contrast, (Tejaswi et al., 2016) reported higher sperm mass activity in Santa Ines sheep fed diets with increasing levels of whole cottonseed, where semen was collected every fourteen days. The discrepancies between studies could be attributed to the varying frequencies of semen collection (Dally et al., 2000).

There was no difference in semen color among the treatments. All semen had a creamy color, which is an indicator of healthy semen, containing a high concentration of spermatozoa (Moghaddam et al., 2012). The results of semen color wasin agreement with the observations of crossbreed rams of Arkhar Merino × Moghani and Baluchi × Moghani rams (Malejane et al., 2014) and Dorper rams (Jibril et al., 2011). The viscosity value observed in the present study was comparable to results from Garut rams (Nasrin et al., 2012) who reported that the semen viscosity was medium-thick.

This study showed no significant effect of supplemental protein and energy on sperm morphology and percentage of sperm abnormality. This agrees with finding of (Kasimanickam et al., 2007) who reported that graded levels of protein in diets had no significant effect on overall sperm morphology of Yankasa rams. The mean percentage normal sperm of this study (91.59) is comparable to the range of 86–98 % reported by (Jiménez-Rabadán et al., 2016). Breeding rams should have >70 % morphologically normal spermatozoa per ejaculation (Asaduzzaman et al., 2021). The percentage of normal sperm was higher than that observed by (Malama et al., 2013) who reported 85.27 % and (Cheah Y. & Yang W, 2011) who reported 78.48 % normal spermatozoa per ejaculation. The percentage of abnormality result in this study was comparable to result of (Nasrin et al., 2012) who reported that spermatozoa abnormality of Garut rams was 7.1 %.

The potential of rams to serve ewes highly dependents on the measure of semen volume, sperm motility, and sperm concentration (Kurnia et al., 2020) which determines the amounts of motile sperm per-ejaculate and prediction in serving females to be higher in supplemented groups than in the control/farmers’ practice. Supplementation of protein above maintenance plays a role in regulating and stabilizing plasma membrane in the process of spermatogenesis (William & Walker, 2011). The current study observed improved (*P* < 0.05) potential of rams to serve ewes when fed protein and energy levels above farmers practice.

The correlations between reproductive parameters in the present study align with the findings reported by (Malejane et al., 2014) in studies on Arkhar Merino × Moghani (AM × MG) and Baluchi × Moghani (BL × MG) rams in Iran. Scrotal circumference and semen volume showed a strong correlation (*r* = 0.89) and were associated with spermatogenesis and testosterone secretion (Maksimović et al., 2016; Bintara et al., 2017). This study aligns with previous reports (Daramola et al., 2007; Dufour et al., 1984) that reported positive and strong correlations, above *r* = 0.85) of SC with semen volume. In this study, correlations between SC and other semen characteristics were low**.**

High reproductive performance of sheep is critical to improve meat production in the farming system. The current study showed that local agro-industrial by-products in Ethiopia are viable option for rams flushing and can be used alongside breeding rams genetic improvement programs. Seasonality will tend to affect nutritional quality of pastures. Semen collected in the dry seasons had higher frequency of spermatozoa abnormalities (Chella et al., 2017). Therefore, further studies are required to assess optimal levels of protein and energy supplementation for enhanced reproductive performance of Menz rams across different seasons.

## 5 Conclusion

This study has demonstrated that short-term nutritional flushing of Menz breeding rams with energy and protein supplementation above what farmers typically feed can improve the rams' body condition, scrotal circumference, semen volume, spermatozoa concentration, and rams’ potential to serve ewes.

## Funding sources

This study was initiated under CGIAR Research Program (CRP) on Livestock with completion under the CGIAR initiative on 'Sustainable Animal Productivity for Livelihoods, Nutrition and Gender inclusion' (SAPLING). The authors acknowledge the financial support from the CRP on Livestock and SAPLING, both supported by contributors to the CGIAR Trust Fund and Amhara Agricultural Research Institute.

## Ethical Statement

The experiment was conducted following guidelines for ethical conduct in the care and use of nonhuman animals in research (CARE 2012).

## CRediT authorship contribution statement

**Tesfa Getachew:** Writing – original draft, Visualization, Validation, Software, Methodology, Investigation, Formal analysis, Data curation, Conceptualization. **Bimrew Asmare:** Writing – review & editing, Visualization, Validation, Supervision, Software, Methodology, Investigation, Formal analysis, Data curation. **Ashraf Alkhtib:** Writing – review & editing, Supervision, Software, Methodology. **Wamatu Jane:** Writing – review & editing, Validation, Supervision, Resources, Project administration, Methodology, Funding acquisition.

## Declaration of competing interest

The authors declare no conflict of interest regarding this study.
